# Small nucleolar RNA 78 promotes the tumorigenesis in non-small cell lung cancer

**DOI:** 10.1186/s13046-015-0170-5

**Published:** 2015-05-15

**Authors:** Di Zheng, Jie Zhang, Jian Ni, Jie Luo, Jiying Wang, Liang Tang, Ling Zhang, Li Wang, Jianfang Xu, Bo Su, Gang Chen

**Affiliations:** Department of Medical Oncology, Shanghai Pulmonary Hospital, Tongji University School of Medicine, 507 Zhengmin Road, Shanghai, 200433 People’s Republic of China; Central Lab, Shanghai Pulmonary Hospital, Tongji University School of Medicine, Shanghai, China; Department of Pathology, Zhongshan Hospital, Fudan University, 180 Fenglin Road, Shanghai, 200032 People’s Republic of China

**Keywords:** Non-small cell lung cancer, Small nucleolar RNA, SNORD78, Epithelial-mesenchymal-transition, Cancer stem-like cell

## Abstract

**Background:**

Accumulating evidence suggests that dysregulated snoRNA may play a role in the development of malignancy. In the present study, we investigated the role of SNORD78 in the tumorigenesis of non-small cell lung cancer (NSCLC).

**Methods:**

We determined the expression level of SNORD78 in NSCLC tissues with quantitative real-time PCR and then studied its clinical significance. We explored the biological significance of SNORD78 with gain-and-loss-of-function analyses both *in vitro* and *in vivo*.

**Results:**

A great upregulation of SNORD78 was observed in cancer tissues compared to their adjacent normal tissues. Meanwhile, patients with high SNORD78 expression have significantly poorer prognosis than those with low expression. Inhibition of SNORD78 suppressed the proliferation of NSCLC cells via inducing G0/G1 cell cycle arrest and apoptosis while SNORD78 overexpression promoted the cell proliferation. SNORD78 promoted invasion of NSCLC cells via inducing epithelial-mesenchymal-transition (EMT). SNORD78 was also obviously upregulated in cancer stem-like cells and is required for the self-renewal of NSCLC. The oncogenic activity of SNORD78 was also confirmed with *in vivo* data.

**Conclusion:**

Our study identified that SNORD78 may be a potential therapeutic target for NSCLC.

## Introduction

Lung cancer represents the most frequent cause of cancer-related mortality [1]. Non-small cell lung cancer (NSCLC), including adenocarcinoma and squamous cell carcinoma, accounts for about 85 % of all lung cancers [2]. Most of NSCLC patients are diagnosed at advanced stages due to the fact that they are usually asymptomatic at early stages [3]. In spite of the recent advances in the management of NSCLC, the prognosis of NSCLC remains relatively poor [4]. Therefore, it is of urgent need to identify novel biomarker of NSCLC and reveal the precise molecular mechanism of the development of NSCLC.

Recently, accumulating literature has demonstrated that noncoding RNAs (ncRNAs), including microRNAs (miRNAs), long noncoding RNAs (lncRNAs) and small nucleolar RNAs (snoRNAs), play an important role in the pathogenesis of NSCLC [5–7]. Small nucleolar RNAs are a group of molecules range between 60–300 nucleotides (nt) in length and represent one of the best characterized groups of ncRNAs [8]. They are prominently found in the nucleolus and functions to guide RNAs for post-transcriptional modification of ribosomal RNAs and some spliceosomal RNAs [9]. From a structural basis, snoRNAs fall into two categories termed box C/D snoRNAs (SNORDs) and box H/ACA snoRNAs (SNORAs) [9, 10]. SNORDs define the target sites for 2’‑O-ribose methylation of rRNAs or snRNAs, whereas SNORAs define the target sites for the isomerization of uridine residues into pseudouridine [10]. A growing volume of literature has indicated that dysregulated snoRNA may play a role in the development of malignancy. For example, Mei et al. [7] demonstrated that SNORA42 acts an oncogene in lung cancer. SNORD33, SNORD66, SNORD73B, SNORD76, SNORD78, and SNORA42 could be potential biomarker of non-small-cell lung cancer [11]. SNORD113-1 functions as a tumor suppressor role in hepatocellular carcinoma [9].

A growing volume of work has proposed that the cancer cell population are heterogeneous, and that a relatively small subpopulation of cancer cells often termed “cancer stem-like cells” (CSCs) or “tumor-initiating cells” harbored enhanced therapy resistance and brisk tumorigenicity [12, 13]. As a result, CSCs are responsible for tumor propagation, resistance to conventional therapy, and tumor recurrence [14]. Mannoor et al. [15] profiled snoRNA expression in cancer stem cells of lung cancer and found that several snoRNAs were significantly upregulated in tumor-initiating cells of lung cancer. We found that snoRD78 was consistently reported to be upregulated in lung cancer [11] and tumor-initiating cells of lung cancer [15], suggesting that SNORD78 might play a role in lung tumorigenesis.

However, the role of SNORD78 in cancer is elusive and few studies have examined its molecular mechanism in NSCLC. In this study, we would like to explore the role of SNORD78 in NSCLC.

## Materials and methods

### Cell culture

Three NSCLC adenocarcinoma cell lines (A549, SPC-A1, NCI-H1975), a NSCLC squamous carcinomas cell line (SK-MES-1), and a normal human bronchial epithelial cell line (16HBE) were purchased from the Institute of Biochemistry and Cell Biology of the Chinese Academy of Sciences (Shanghai, China) and were authenticated. The cell lines were cultured in DMEM or RPMI 1640 (Gibco BRL), containing 10 % fetal bovine serum (FBS, HyClone) as well as 100 U/ml penicillin and 100 μg/ml streptomycin (Invitrogen). Cells were maintained in a humidified incubator at 37 °C in the presence of 5 % CO_2_. All cell lines have been passaged for fewer than 6 months.

### Patient and clinical samples

The human specimens in this study were sanctioned by the local ethics committee of Pulmonary Hospital at Tongji University (Shanghai, China). A total of 56 primary cancer tissues and paired adjacent noncancerous tissues (>5 cm from tumor) were obtained from patients who underwent surgery at Pulmonary Hospital between 2008 and 2011 and were diagnosed with NSCLC (stage II, III, and IV) based on histopathological evaluation. These tissues were flash frozen in liquid nitrogen immediately after surgery and subsequently stored at −80 °C prior to RNA isolation and qRT-PCR analysis. No patients received anticancer treatments before surgery in this study. Complete clinicopathological data of the patients from which the specimens were collected were available. Overall survival (OS) was defined as the interval between the dates of surgery and death.

### RNA extraction and quantitative real-time PCR

Total RNA was isolated from tumor tissues and purchased cell lines using TRIzol (Life Technologies) according to supplier’s instructions. RNA concentration was measured with a NanoDrop ND-2000 spectrophotometer (Life Technologies). Reverse transcription was performed with random primers using the First Strand cDNA synthesis kit (Takara, Otsu, Shiga, Japan) according to the manufacturer’s instructions. The quantitative real-time polymerase chain reaction (qRT-PCR) was performed using the SYBR Select Master Mix (Applied Biosystems, cat: 4472908) on ABI 7500 system (Applied Biosystems, CA, USA) according to the manufacturer’s instructions. β-actin was measured as an internal control. The relative expression fold change of mRNAs was calculated by the 2^−ΔΔCt^ method. After the reverse transcription, 0.5 μl of the complementary DNA was used for subsequent qRT-PCR reaction. The primer sequences were as follows: β-actin: 5’-GAAATCGTGCGTGACATTAA-3’ (forward), 5’-AAGGAAGGCTGGAAGAGTG-3’ (reverse); GAS5: 5’-TGAAGTCCTAAAGAGCAAGCC-3’ (forward), 5’-ACCAGGAGCAGAACCATTAAG-3’ (reverse); SNORD78: 5’-GTGTAATGATGTTGATCAAATGTCTGAC-3’ (forward), 5’-CACATTACTACAACTAGTTTACAGACTGG-3’ (reverse).

For cell expression and tumor samples, each sample was run in triplicate. qRT-PCR results were analyzed and expressed relative to CT (threshold cycle) values, and then converted to fold changes.

### Plasmid, shRNA and cell transfection

Small interfering RNAs targeting GAS5 were bought from Genepharm Technologies (Shanghai, China). Expression vectors encoding SNORD78 were purchased from Fulen Gen Company (Guangzhou China). For stable knockdown of SNORD78, NSCLC cells were transfected with lentiviral constructs encoding two different SNORD78 shRNAs or nontargeting shRNAs in the presence of polybrene (8 mg/mL). After 48 h, transduced cells were grown in culture media containing 1 mg/mL puromycin for two weeks. The shRNA sequences were as the followings: shRNA1: GTTGATCAAATGTCTGACCTG,shRNA2: GACCTGAAATGAGCATGTAGA. For construction of lentiviral vector expressing human SNORD78 gene, SNORD78 cDNA was amplificated by PCR with the primers (forward, 5’-ggggtaccGTGTAATGAT-3’, and reverse, 5’-ccgctcgagTGTAGACAAAGGTA-3’). To obtain cell lines stably expressing SNORD78, A549 cells were infected with the LV-SNORD78 and LV-control viruses. The stable over-expressing cell lines were identified using real-time PCR and named as A549 LV-SNORD78 clone 1 and LV-SNORD78 clone2.

All transfected cells were selected with puromycin (1 mg/ml) for two weeks.

### Cancer cell invasion assay

Cancer cell invasion assay was performed using Boyden chambers with filter inserts (pore size, 8.0 μm, Millipore, MA) coated with Matrigel (40 μl, Sigma, USA) in 24-well dishes (Corning). Briefly, 1−2 × 10^5^ cells after transfected with SNORD78 shRNA or control shRNA, LV-SNORD78 or LV-control were seeded in the upper chamber, while the culture medium (Invitrogen) with 10 % FBS was placed in the lower chamber. The plates were incubated for 24 h or 48 h. The cells on the upper surface were scraped and washed away, whereas the cells on the lower surface were fixed in 4 % formaldehyde and stained with 0.05 % crystal violet for 2 h. Finally, cells were counted under a microscope and the relative number was calculated. Experiments were independently repeated in triplicate.

### Cell viability assay

Cell viability was assessed by the Cell Counting Kit 8 (CCK-8, Donjindo). Briefly, control and treated A549 and H1975 cells were seeded into 96-well plates at an initial density of 3000 and 5000 cells/well. At each time points, 10 μl of CCK-8 solution was added to each well and incubated for 2 h. The absorbance was measured by scanning with a microplate reader at 450 nm. Data were expressed as the percentage of viable cells as follows: relative viability (%) = (A450_treated_−A450_blank_)/(A450_control_ - A450_blank_) × 100 %.

### Cell cycle analysis

Cells transiently transfected with desired vector were harvested 48 h after transfection. The cells were washed for three times by cold PBS, and then cells were fixed in 70 % ethanol in PBS at −20 °C for 24 h. After fixation, cells were washed with cold PBS and stained with 0.5 ml of propidium iodide (PI) staining buffer, which contains 200 mg/ml RNase A, 50 μg/ml PI, at 37 °C for 30 min at 4 °C in the dark. The cell-cycle profiles were assayed at 488 nm on an EPICS 752 flow cytometer (Coulter, Hialeah, FL) equipped with MPLUS software (Phoenix 140 Flow Systems, San Diego, CA). Data were expressed as percentage distribution of cells in G0/G1, S and G2/M phases of the cell cycle.

### Western blot analysis

The harvested cells were centrifuged at 2,000 rpm for 5 min. The total cellular proteins were prepared using RIPA cell lysis buffer (Cell Signaling Technology) supplemented with protease inhibitors. The lysates were then collected and subjected to ultrasonication and centrifugation. The supernatants were collected, and protein content was determined by Bradford assay. Equal amounts (30–50 μg) of proteins were applied to an 8–12 % SDS-polyacrylamide separating gel and transferred to a PVDF Immobilon-P membrane (Millipore). The membrane was blocked with 5 % nonfat milk in TBST and then probed with indicated primary antibodies with gentle shaking at 4 °C overnight. The membranes were washed with TBST (3 × 10 min), incubated in secondary antibodies at room temperature for 1 h. Antibody-bound proteins were detected by BeyoECL Plus kit.

The primary antibodies used in these experiments include E-cadherin (1:1000) (Abcam), N-cadherin (1:1000) (Abcam), Vimentin (1:500) (Abcam), p21 (1:1000), p16 (1:1000) and caspase-3 (1:1000). HRP-conjugated goat anti-rabbit IgG antibody (Abcam) was used as the secondary antibody.

### Immunofluorescence analysis

Cells transfected with desired vector were cultured and fixed on 12 × 12 mm glass slides. For membrane staining (E-cadherin), cells were fixed with cold 100 % methanol for 10 min. For intracellular staining (Vimentin, N-cadherin), the cells were fixed with 4 % (wt/vol) paraformaldehyde in PBS and permeabilized by incubation with 0.5 % Triton X−100 in PBS for 2 min. The cells were incubated with 5 % bovine serum albumin in PBS for 20 min at room temperature. After washing with PBS, the cells were incubated with specific primary antibody at 4 °C overnight. Antibody dilutions of 1:400 were used for E-cadherin (Abcam), N-cadherin (Abcam) and Vimentin (Cell Signaling Technology). The cells were then washed and incubated with Alexa Fluor 488-conjugated goat anti-rabbit IgG for 1 h. The nuclei were stained with 4,6-diamidino-2-phenylindole (DAPI). Sections were visualized by a LEICA DMI 3000B fluorescence microscope with an original magnification × 200.

### Mammosphere-formation assay

Single cells were plated in ultralow-attachment six-well plate (Corning) at a density of 10,000 viable cells/mL. Cells were grown in a serum-free DMEM/F12 medium (Invitrogen), supplemented with 20 ng/mL EGF and 20 ng/mL bFGF (BD Biosciences). Bovine pituitary extract was excluded. Fresh medium were added 0.5 ml/well every three days. Mammospheres >50 μm in diameter were counted at day 8. Images were taken with a LEICA DMI 3000B with original magnification × 100 or × 400.

### Methylation-specific polymerase chain reaction

The methylation status of the E-cadherin promoter region was determined by methylation-specific PCR (MSP) using bisulfite-modified DNA. Genomic DNA was extracted using the QIAamp DNA mini kit (Axygen). Two primer sets were used to amplify the promoter region of the E-cadherin that incorporated a number of CpG sites, one specific for the methylated sequence (E-cadherin-M, forward: 5’-TATGAGTTGTAAGCGGTAGAGTTC-3’; reverse: 5’-TACGAACTTAACGAAAAAAAATCAT-3’) and the other for the unmethylated sequence (E-cadherin-U, forward: 5’- GAATATGAGTTGTAAGTG GTAGAGTTT-3’; reverse: 5’-TACAAACTTAACAAAAAAAAATCATACT-3’). The primers used in the present study detect specifically the promoter sequence of the E-cadherin. M and U are the PCR products of methylated and unmethylated alleles, respectively. The polymerase chain reactions for E-cadherin-M and E-cadherin-U were carried out in a 50 μl volume containing 1× polymerase chain reaction buffer (15 mmol/l MgCl_2_), 2.5 mmol/l mixture of dNTPs, 10 pMof each primer, 4 U HotStart Taq DNApolymerase (Qiagen, Frankfurt, Germany), and 25 ng to 50 ng of bisulfitemodified DNA. Amplification was performed in a thermocycler with the following conditions: 94 °C for 3 min, cycled at 94 °C for 15 s, 60 °C or 57 °C for 15 s, and 72 °C for 15 s (35 cycles), followed by an extension at 72 °C for 7 min. Methylation-specific PCR experiments were performed at least in duplicate.

### Animal experiments

Briefly, male BALB/cathymic nude mice (4–6 weeks old) were obtained from the experimental animal center of Shanghai Institute for Biological Science. To evaluate the effect of SNORD78 in nude mice, we constructed NSCLC H1975 cells with SNORD78 stable down expression. All mice were injected subcutaneously into either side of flank area with 1 × 10^7^ SNORD78 stablely downregulated or control H1975 cells to establish the NSCLC xenograft model. Tumor volumes were measured (0.5 × length × width^2^) in mice on a weekly basis. After 6 weeks, mice were sacrificed, and tumors were exercised and subjected to immunohistochemical analysis of Ki67. All animal experiments were performed in animal laboratory center of Pulmonary Hospital and in accordance with the Guide for the Care and Use of Laboratory Animals published by the US National Institutes of Health (NIH publication no. 85–23, revised 1996). The study protocol was approved by the Animal Care and Use committee of Pulmonary Hospital.

### Statistical analysis

All statistical analyses were performed with SPSS version 19.0 software (SPSS software, Armonk, NY, USA). All data were presented as mean ± standard deviation from three independent experiments with each measured in triplicate. The gene expression level of SNORD78 in tumors was compared with adjacent normal tissues utilizing the paired sample *t*-test, whereas the association between SNORD78 expression and clinical characteristics was evaluated using the chi-square test. Survival curves were plotted by the Kaplan-Meier method, and the long-rank comparison was carried out to assess differences between stratified survival groups using the median value as the cutoff. A Cox proportional hazards analysis was performed to calculate the hazard ratio (HR) and the 95 % confidence interval (CI) to evaluate the association between SNORD78 expression and survival. Cox regression analysis was used to determine the independent factors, which were based on the variables selected by univariate analysis. The expression differences between cell lines, the expression changes after transfection, cell viability and cell invasion assays were analyzed using independent samples *t*-test.

## Results

### Characterization of SNORD78 in NSCLC

**Fig. 1:**
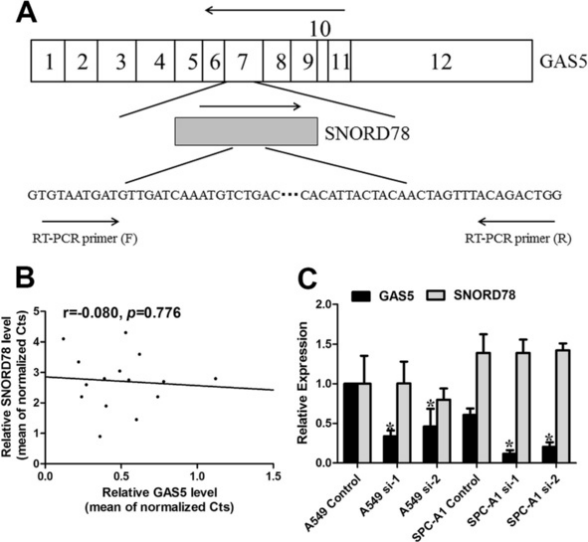
SNORD78 is transcribed independently of GAS5. (**a**) Schematic diagram of SNORD78 and its location in GAS5. GAS5 (top) consists of 12 exons and SNORD78 resides in intron 7–8 of GAS5. SNORD78 is depicted as the gray box. The location of the forward (F) and reverse (R) primers used for RT-PCR are shown. Orientation of arrows indicates the direction of the transcription or amplification reaction. (**b**) Linear correlation between the expression of the SNORD78 and GAS5 was not observed. ΔCt values were used to measure gene expression, which was normalized by β-actin expression levels. (**c**) Expression of GAS5 was decreased, and SNORD78 was not significantly changed in A549 and SPC-A1 cells with two different siRNAs against GAS5. Statistical differences were analyzed relative to siRNA control. *, *p* < 0.05

SNORD78 is located in 1q25, one of most frequently amplified chromosomal segments in solid tumors, particularly NSCLC [16–18]. Furthermore, we performed a computational screen and found that SNORD78 resides in intron 7–8 of GAS5 (Fig. 1a). To explore the potential relationship between the SNORD78 and GAS5 transcripts, we first examined the expression levels of SNORD78 and GAS5 in 15 NSCLC tissues. The results showed that no correlation (r = −0.080, *p* = 0.776) existed between the transcriptional levels of SNORD78 and GAS5 (Fig. 1b). Furthermore, SNORD78 was statistically unchanged in A549 and SPC-A1 cells transfected with two different short interfering (siRNAs) (designated as si-1 and si-2) against GAS5, despite significant reduction in GAS5 messenger RNA expression (Fig. 1c).

### SNORD78 was upregulated in NSCLC

**Fig. 2:**
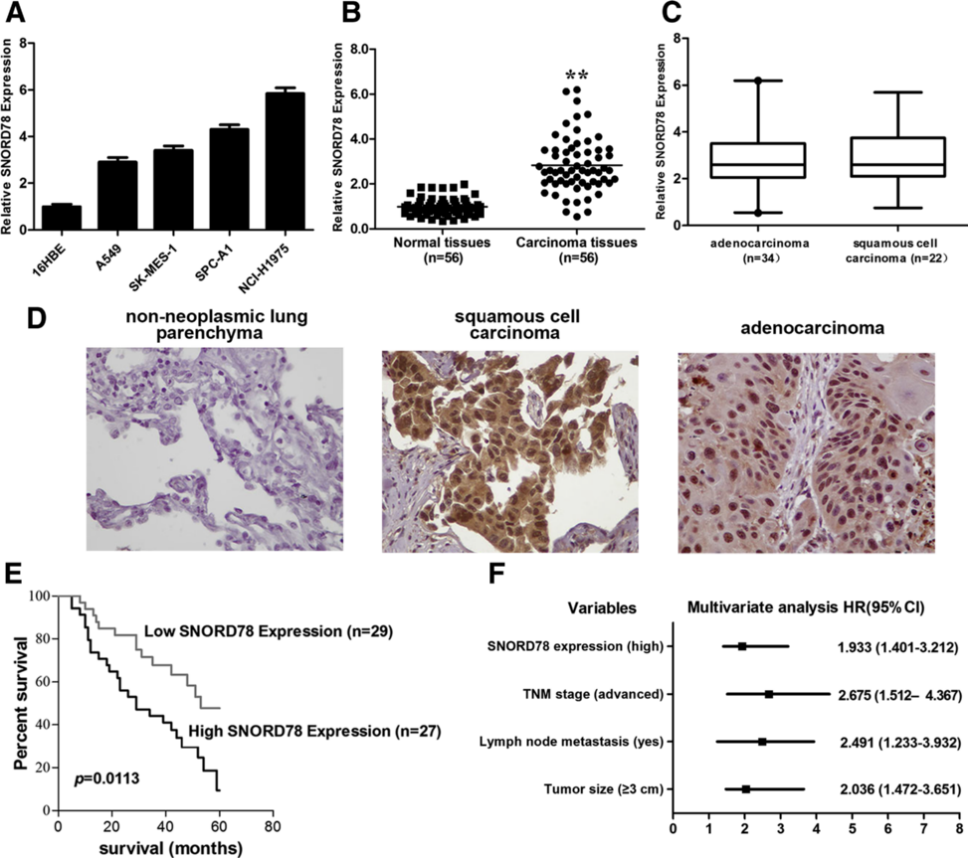
SNORD78 expression in NSCLC cell lines, cancer tissues and its clinical significance. (**a**) qRT-PCR analysis of SNORD78 expression levels in NSCLC cell lines (A549, SK-MES-1, SPC-A1 and NCI-H1975) compared with the normal bronchial epithelial cell line (16HBE). (**b**) Difference in expression levels of SNORD78 between non-small cell lung cancer tissues and matched non-tumor NSCLC tissues. The expression of SNORD78 was normalized to β-actin. The statistical differences between samples were analyzed with paired samples *t*-test (n = 56, p < 0.0001). (**c**) There were no significant differences in expression levels of SNORD78 between adenocarcinoma tissues and squamous cell carcinoma tissues. The expression of SNORD78 was normalized to β-actin. The statistical differences between samples were analyzed with paired samples *t*-test (p = 0.9577). (**d**) Negative staining of SNORD78 in adjacent non-neoplasmic lung parenchyma. Positive mainly nuclear expression of SNORD78 in representative cases of squamous cell carcinoma and adenocarcinoma. (magnification × 400) (**e**) Patients with high levels of SNORD78 expression showed reduced overall survival times compared with patients with low levels of SNORD78 expression (p =0.0113, log-rank test). *, *p* < 0.05; **, *p* < 0.01. (**f**) Multivariate analysis of HRs for OS is shown. HRs are presented as the means (95 % CI)

SNORD78 was reported to be upregulated in lung cancer tissues [11]. To further corroborate the previous finding of Liao et al. [11], we firstly detected SNORD78 expression in a series of lung cancer cell lines including adenocarcinoma and squamous carcinoma subtypes utilizing qRT-PCR. When normalized to normal bronchial epithelial cell line (16HBE), the RNA level of SNORD78 was increased in the NSCLC cancer cell lines (A549, SK-MES-1, SPC-A1, NCI-H1975) by 2.7- to 5.8-fold compared with 16HBE (Fig. 2a).

We detected SNORD78 expression in 56 pairs of NSCLC tissues and in their adjacent normal tissues utilizing qRT-PCR. The data showed that SNORD78 expression was obviously increased, and the average intensity was increased 3-fold in tumorous than in adjacent normal tissues (Fig. 2b). The average ΔCt expression value of SNORD78 was 6.75 in NSCLC tissues, ranking from 5.91 to 8.32, when compared with β-actin. Furthermore, there were no statistical differences of expression of SNORD78 between adenocarcinoma and squamous cell carcinoma, suggesting that SNORD78 was shared in two major histological types of NSCLC (Fig. 2c). In situ hybridization (ISH) studies confirmed the upregulation of SNORD78 in NSCLC (Fig. 2d).

### The association between SNORD78, clinicopathological characteristics and prognosis after surgery

The patients with NSCLC were then stratified according to SNORD78 expression level (median split) and compared for different clinicopathologic features (age, sex, tumor size, lymph node status, distant metastasis, and survival time). Results revealed that SNORD78 expression was positively correlated with the TNM stage (*p* = 0.0354, *p* < 0.05), lymph node metastasis (*p* =0.0004, *p* < 0.05) and tumor size (*p* =0.024, *p* < 0.05). There was no significant correlation between SNORD78 expression with age, gender, or degree of differentiation (Table 1).

**Table 1:** Correlation between SNORD78 expression and clinicopathologic characteristics

	SNORD78 expression			
Clinicopathologic parameters	High	Low	Number of cases	*p* value
Age (years)	10	14	24	0.2301
≤60	14	18	32	
>60				
Gender				
Male	13	11	24	0.4043
Female	13	19	32	
Diameter				
≤3	11	9	20	0.024^a^
>3	24	12	36	
Lymph node metastasis				
Yes	21	9	30	0.0004^a^
No	13	13	26	
TNM stage				
I–II	5	16	21	0.0354^a^
III	25	10	35	
Degree of differentiation				
Well and moderately	14	16	30	0.3778
Poorly	15	11	26	

^a^Statistically significant (*p* < 0.05)

Kaplan-Meier survival analysis and log-rank tests using patient postoperative survival were conducted to further evaluate the correlation between SNORD78 and prognosis of patients with NSCLC. The cumulative survival rate was significantly better in patients with lower-SNORD78-expressing tumours than in those with higher- SNORD78-expressing tumors (*p* = 0.0113, HR (95 % CI) = 1.933 (1.401-3.212)); Fig. 2d). In addition, the univariate analysis revealed that the tumor size, lymph node metastasis, TNM stage and SNORD78 expression were significantly correlated with overall survival (Table 2). The multivariate analysis demonstrated that tumor size (*p* = 0.011), lymph node metastasis (*p* = 0.015), TNM stage (*p* = 0.001) and SNORD78 expression (*p* = 0.007) were independent risk factors for overall survival (Fig. 2e) (Table 2).

**Table 2:** Univariate and multivariate analysis of factors associated with overall surivival in NSCLC patients (56 pairs)

	Univariate analysis		Multivariate analysis	
Risk factors	HR (95 % CI)	*p* value	HR (95 % CI)	*p* value
Age (≤60/>60)	2.899 (0.997–8.426)	0.063		
Gender(male/female)	3.618 (0.948–13.812)	0.06		
Diameter(≥3/<3)	3.125 (1.093–8.929)	0.035^a^	2.036 (1.472–3.651)	0.011^a^
Lymph node metastasis(Yes/No)	3.703 (1.119–11.507)	0.014^a^	2.491 (1.233–3.932)	0.015^a^
TNM stage (III/I–II)	4.689 (1.633–14.506)	0.030^a^	2.675 (1.512–4.367)	0.001^a^
SNORD78 expression(high/low)	5.495 (1.678–17.857)	0.005^a^	1.933(1.401–3.212)	0.007^a^
Histological grade (poorly/well and moderately)	1.666 (0.652–4.259)	0.408		

^a^Statistically significant (*p* < 0.05)

### SNORD78 is required for efficient proliferation of NSCLC cells

**Fig. 3:**
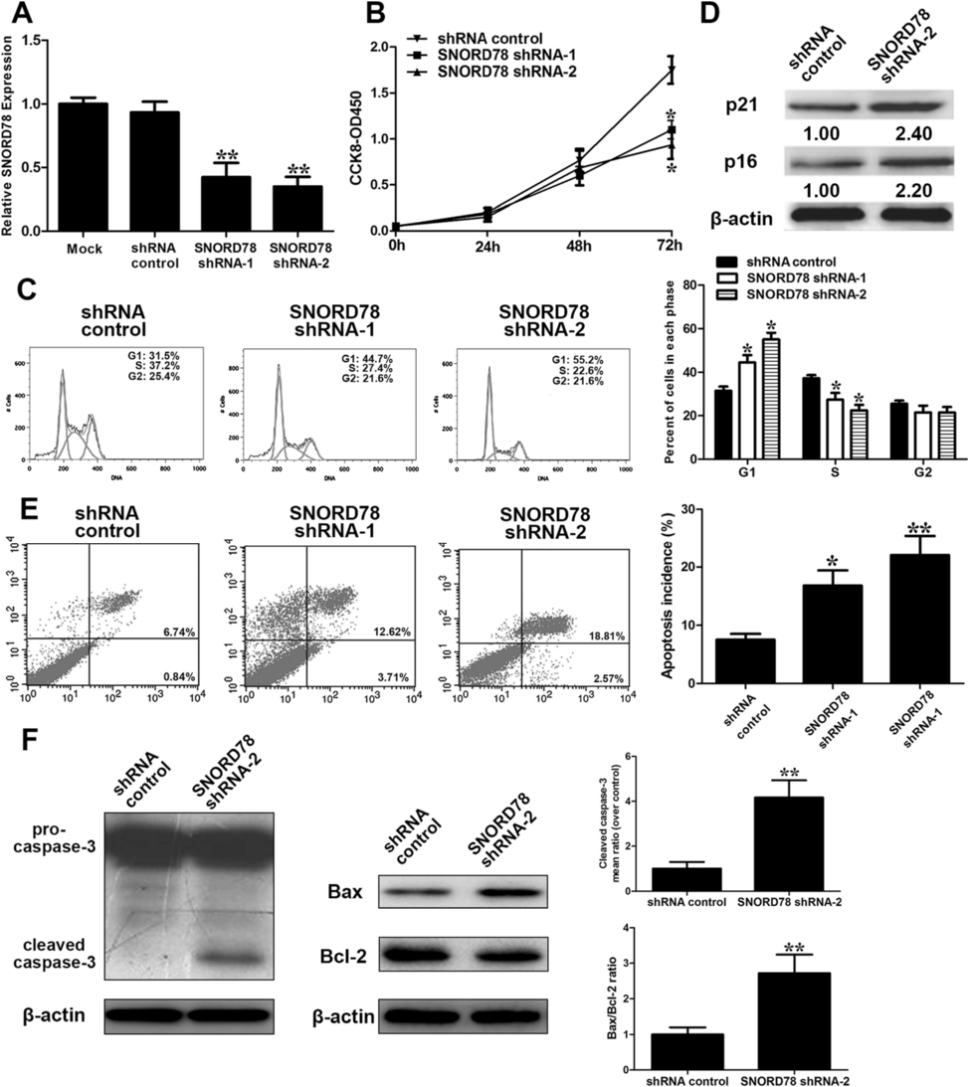
Effect of SNORD78 knockdown on NSCLC cell viability, cell cycle and apoptosis. (**a**) qRT-PCR analysis of SNORD78 expression following treatment of H1975 cells with two specific shRNAs targeting SNORD78. (**b**) H1975 cells were transfected with SNORD78 shRNAs or shRNA control. CCK8 assays were performed to determine the proliferation of H1975 cells. (**c**) Cell cycle analysis determined the relative cell numbers in each cell-cycle phase after propidium iodide staining of SNORD78-downregulated H1975 cells. Numbers inside bars represent percentages of cells in each phase. Data represent the mean ± S.D. from three independent experiments. (**d**) H1975 cells, treated as described in Fig. 3a, were collected for western blotting analysis of the G0/G1 arrest markers, p16 and p21. Relative protein expression was identified (n = 3). (**e**) Annexin V/PI staining and flow cytometry analysis assessing apoptosis in H1975 cells after SNORD78 shRNAs transfection. Data represent the mean ± S.D. from three independent experiments. (**f**) Changes in pro-apoptotic factor caspase-3 activation and Bax/Bcl-2 ratio were shown by western blotting analysis and normalized to β-actin after SNORD78 shRNAs transfection. Data represent the mean ± S.D. from three independent experiments. *, *p* < 0.05; **, *p* < 0.01

The fact that SNORD78 is frequently upregulated in primary NSCLC tissues and NSCLC cells prompted us to explore the role of SNORD78 dysregulation in NSCLC cells. We constructed cell lines that stably downregulated SNORD78 by lentivirus infection of H1975 cells with specific shRNAs (Fig. 3a), which expressed relatively higher level of SNORD78. The CCK-8-based assay detected significant decrease in the proliferation of H1975 cells after knockdown of SNORD78 (Fig. 3b). Thus, we evaluated the effect of SNORD78 expression on cell cycle distribution and cell apoptosis in H1975 cells by flow cytometry. We found that SNORD78 silencing induced a significant G0/G1 arrest (Fig. 3c). Furthermore, the western blot analysis indicated that knockdown of SNORD78 significantly increased the expression of G0/G1 arrest markers, p16 and p21 (Fig. 3d). These data demonstrated that SNORD78 silencing induced H1975 cell cycling arrest at the G0/G1 phase. Flow cytometry assay indicated that suppression of SNORD78 induced the increase of cell apoptosis by phycoerythrin (PE)-conjugated Annexin V staining and FACS (Fig. 3e). To explore the underlying mechanism of SNORD78’s role in cell apoptosis, we determined the expression of pro-apoptotic factor, Bax, Bcl and caspase-3 by western blot. SNORD78 knockdown increased the expression levels of cleaved caspase-3 and Bax/Bcl-2 ratio (Fig. 3f). These results demonstrated that SNORD78 knockdown may inhibit the proliferation of NSCLC cells through inducing cell G0/G1 arrest and apoptosis.

**Fig. 4:**
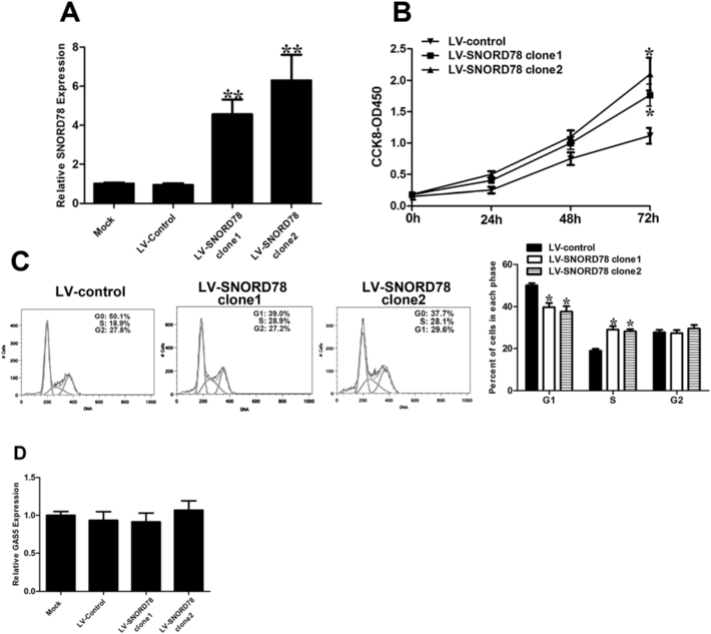
Effect of SNORD78 overexpression on NSCLC cell viability and cell cycle. (**a**) qRT-PCR analysis of SNORD78 expression following transfection of A549 cells with SNORD78. (**b**) A549 cells were transfected with SNORD78 or control. CCK8 assays were performed to determine the proliferation of A549 cells. (**c**) Cell cycle analysis determined the relative cell numbers in each cell-cycle phase after propidium iodide staining of SNORD78 overexpressed A549 cells. Numbers inside bars represent percentages of cells in each phase. (**d**) qRT-PCR analysis of GAS5 expression following transfection of A549 cells with SNORD78. Data represent the mean ± S.D. from three independent experiments. *, *p* < 0.05; **, *p* < 0.01

To confirm the effect of ectopic expression of SNORD78 on cell proliferation, we overexpressed SNORD78 by lentivirus infection (Fig. 4a). The results showed that the overexpression of SNORD78 increased proliferation of NSCLC cancer cell *in vitro* (Fig. 4b,c). To exclude the possibility that biological effects of SNORD78 depend on its regulation of GAS5, we determined the expression level of GAS5 with SNORD78 overexpression. We found that overexpression of SNORD78 had no significant effects on the expression level of GAS5 (Fig. 4d). These finding suggest that SNORD78 may take a part in the development of NSCLC.

### SNORD78 promoted invasion of NSCLC cells via inducing epithelial-mesenchymal-transition (EMT)

**Fig. 5:**
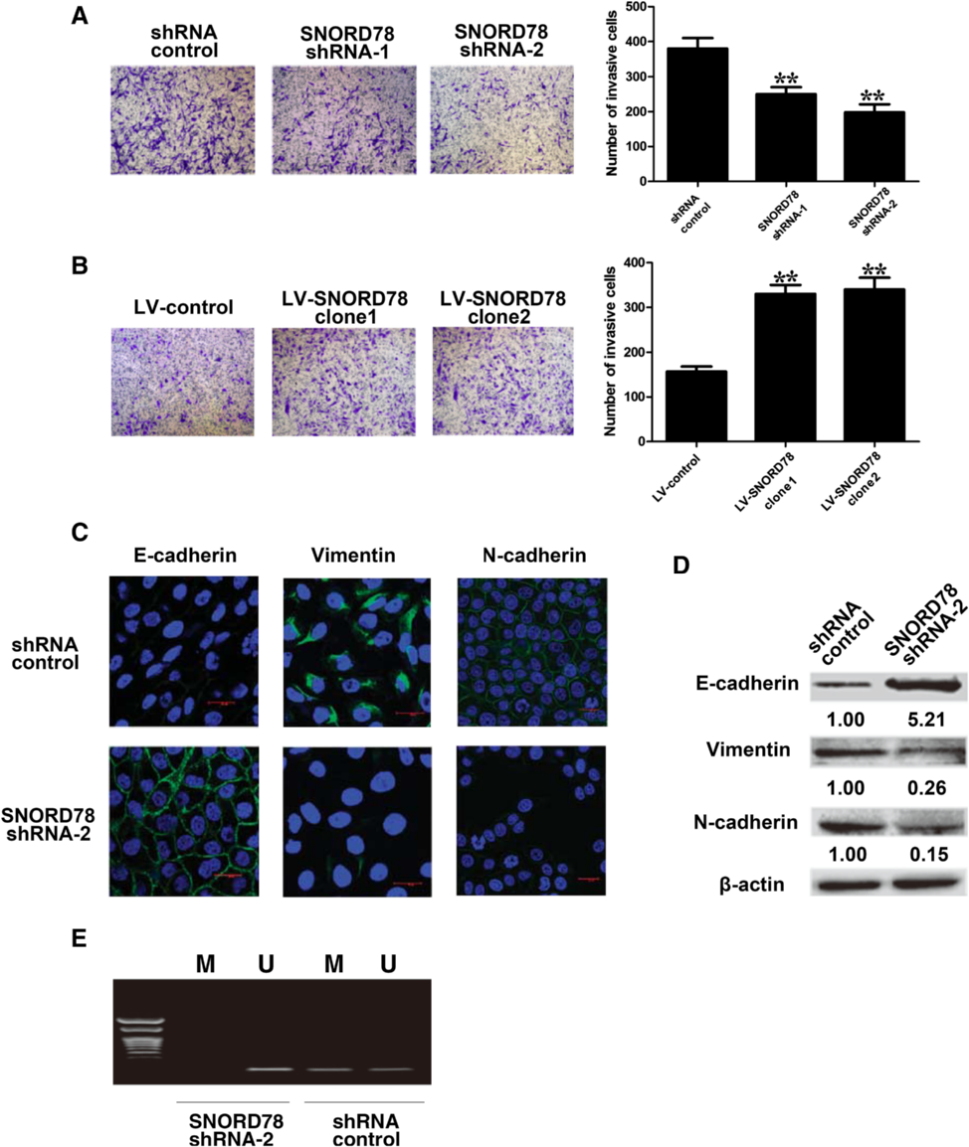
SNORD78 promoted invasion of NSCLC cells via inducing epithelial-mesenchymal-transition (EMT). (**a**) H1975 cells were transfected with shRNA control or shRNA SNORD78. Transwell assays were performed to investigate the invasive ability of H1975 cells. Data represent the mean ± S.D. from three independent experiments. (**b**) A549 cells were transfected with LV-control or LV-SNORD78. Transwell assays were performed to investigate the invasive ability of A549 cells. Data represent the mean ± S.D. from three independent experiments. *, *p* < 0.05; **, *p* < 0.01. (**c**) immunofluorescence images of H1975 cells stained using antibodies against E-cadherin, Vimentin or N-cadherin after transfected with shRNA control or SNORD78 shRNA-2 (original magnification × 200). (**d**) Western-blot analysis of phenotypic markers after SNORD78 knockdown in H1975 cells. Relative protein expression was identified (n = 3) and normalized to β-actin. Data represent the mean ± S.D.. (**e**) Results of MSP analysis of E-cadherin gene in SNORD78 shRNA-2-transfected H1975 cells, and control cells. M and U, PCR products of methylated and unmethylated alleles, respectively

Transwell assay revealed a substantial decrease in the number of cells that penetrated the porous filter with SNORD78 knockdown, suggesting impaired invasion ability of H1975 cells (Fig. 5a). Meanwhile, a significant increase in cancer cell invasion was observed in NSCLC cells A549 *in vitro* with SNORD78 overexpression (Fig. 5b). These data suggest that SNORD78 promoted the invasion of NSCLC cells. Invasion is an important characteristic of NSCLC and emerging evidence has linked invasion with EMT. The epithelial-mesenchymal-transition (EMT) is a well-coordinated process that occurs during embryonic development and a pathological feature in tumorigenesis [19, 20]. During such a process, the epithelial phenotype cells lose the expression of E-cadherin and other components of cell to cell junctions and adopt a mesenchymal phenotype [21]. The EMT process has been shown to play a vital role in cancer invasion, metastasis, expansion of the population of cancer stem cells and therapeutic resistance [21]. We then examined the effect of SNORD78 on the EMT process of NSCLC cells.

Compared with the vector-transfected cells, H1975 cells became more round-shaped and came up with an epithelial phenotype with SNORD78 knockdown (Fig. 5c). With the immunofluorescence analysis, inhibition of SNORD78 in H1975 cells resulted in an obvious upregulation in the expression of epithelial marker E-cadherin and a great reduction in the expression of mesenchymal markers N-cadherin and Vimentin (Fig. 5c). The western blot analysis confirmed the results from the immunofluorescence analysis (Fig. 5d). Together, it indicates that SNORD78 promoted the EMT process and thus, SNORD78 might play a role in the development of NSCLC.

As SNORDs are known to define the target sites for 2’‑O-ribose methylation of rRNAs or snRNAs, we explored whether DNA methylation contributes to the EMT process with Methylation-Specific PCR to examine the role of methylation in deregulation of E-cadherin. We found that the promoter regions of E-cadherin gene from control H1975 cells were strongly methylated, whereas the SNORD78 silenced H1975 cells had unmethylated E-cadherin promoter region (Fig. 5e). These results indicate that aberrant methylation of E-cadherin gene promoter by SNORD78 may contribute to the EMT process in NSCLC.

### SNORD78 is required for the self-renewal of cancer-stem cells of NSCLC

**Fig. 6:**
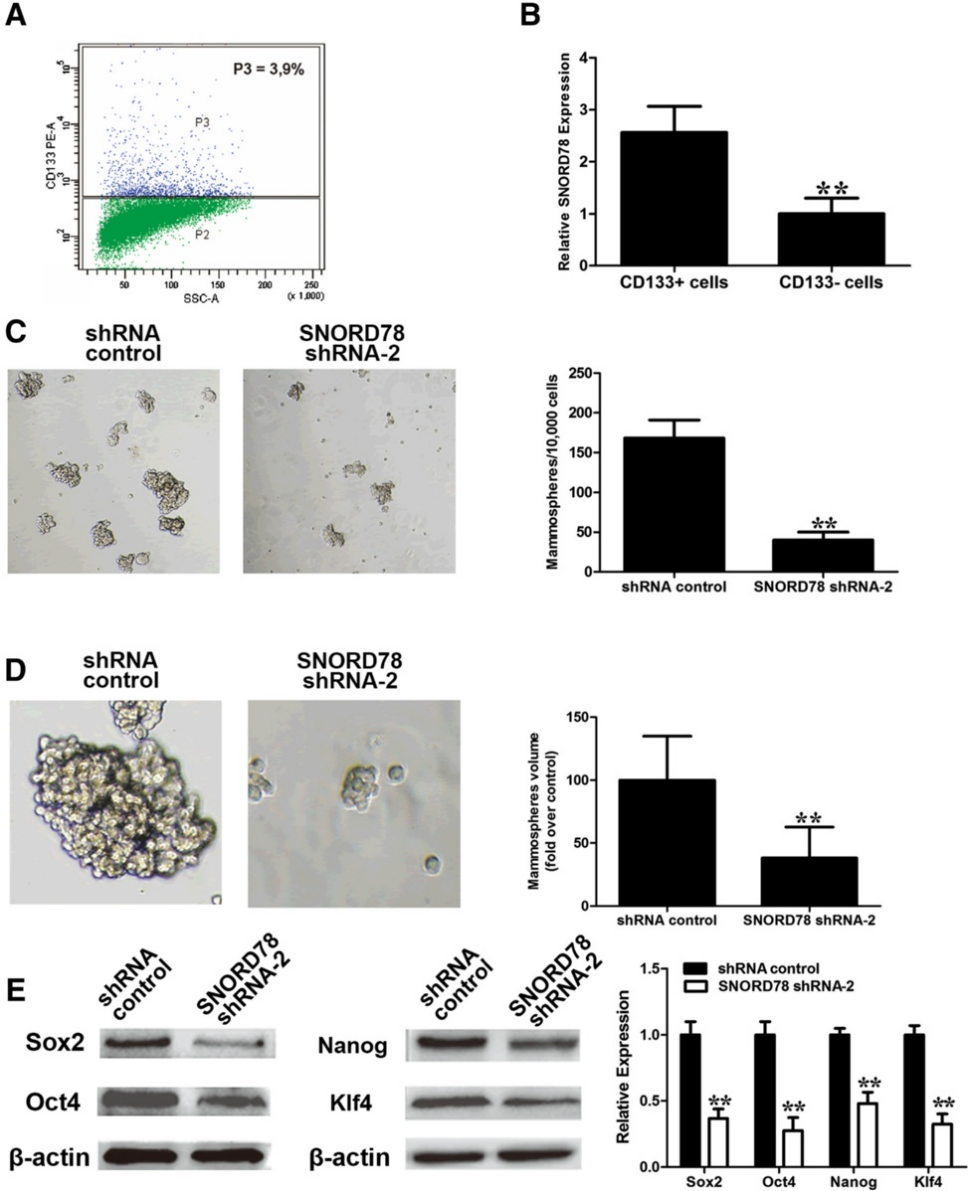
SNORD78 is required for the self-renewal of cancer-stem cells of NSCLC. (**a**) CD133 positive cells were isolated from NSCLC cells A549. (**b**) There was a high level of expression of SNORD78 in CD133+ cells versus CD133- cells of A549 cells as determined by qPCR analysis. (**c**) Representative images of mammospheres formed from A549 cells. Inhibition of SNORD78 reduced the number of mammospheres (original magnification × 100). (**d**) Smaller sizes of mammospheres were observed after SNORD78 knockdown in comparison with control (original magnification × 400). Representative images of mammospheres were shown above. Experiments were independently repeated at least three times. (**e**) Western-blot analysis of stemness factors after SNORD78 knockdown in H1975 cells. Relative protein expression was identified (n = 3) and normalized to β-actin. Data represent the mean ± S.D..*, *p* < 0.05; **, *p* < 0.01

Mannoor et al. [15] profiling results revealed SNORD78 was upregulated in cancer-stem cells of NSCLC. We used CD133^+^ and CD133^−^cells that were isolated from A549 cells (Fig. 6a) and confirmed that SNORD78 was specifically upregulated in cancer-stem cells (Fig. 6b). Furthermore, shRNA-SNORD78 transfected cells formed fewer (Fig. 6c) and smaller mammospheres (Fig. 6d) compared with vector-transfected cells, implying that SNORD78 is required for the self-renewal of cancer-stem cells of NSCLC. Furthermore, inhibition of SNORD78 resulted in the downregulation of a series of stemness factors, which has been shown to play an important in the self-renewal of cancer stem-like cells in NSCLC [22, 23].

### SNORD78 knockdown inhibits *in vivo* tumorigenesis of NSCLC cells

**Fig. 7:**
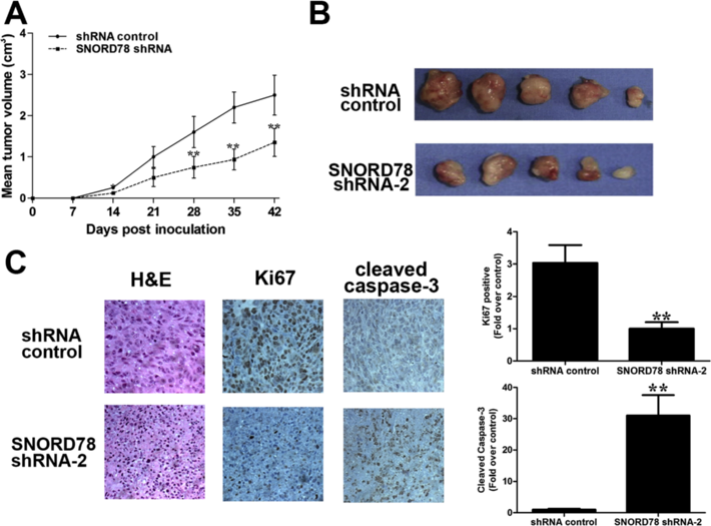
The effects of SNORD78 on *in vivo* tumor growth of NSCLC. Inhibition of SNORD78 suppressed tumor growth in subcutaneous implantation mouse models of H1975 cells. Tumor growth curves (**a**) and tumor volumes (**b**) of subcutaneous implantation models of gallbladder cancer are shown. (**c**) H&E and immunohistochemical staining demonstrated that suppression of SNORD78 inhibited the aggressive phenotype of NSCLC cells *in vivo*, as indicated by the expression of Ki67-positive and caspase-3-positive cells. *, *p* < 0.05; **, *p* < 0.01

To validate the effect of SNORD78 on NSCLC cell tumorigenesis *in vivo*, H1975-shRNA-SNORD78 or H1975-shRNA-control cells were injected into flanks of nude mice. Palpable tumors formed with 1 week. Tumor volume was measured on a weekly basis. Four weeks after injection, the average tumor volume of H1975 cells transfected with shRNA-SNORD78 was 1.35 ± 0.34 cm^3^, which was significantly lower than tumors in the control group (2.51 ± 0.48 cm^3^, *p* < 0.01; Fig. 7a,b). Immunohistochemical staining of tumor tissues indicated a decrease in ki67 and an increase in cleaved caspase-3 in shRNA-SNORD78 vs. shRNA-control (Fig. 7c). The *in vivo* data complement the *in vitro* studies of SNORD78 and confirm the oncogenic activity of SNORD78 in NSCLC.

### Predictive value of SNORD78

**Fig. 8:**
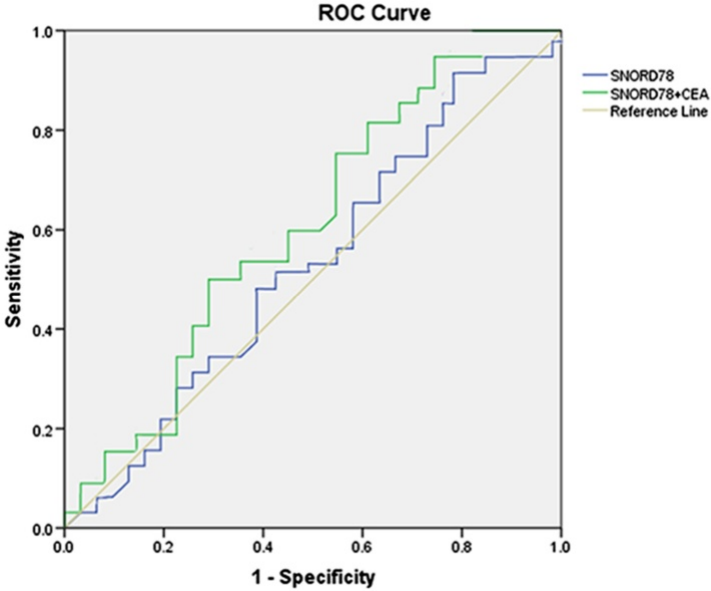
SNORD78 might be a biomarker in NSCLC. Expression of SNORD78 was detected in subgroups grouped by metastasis. Further ROC curve analysis was used for merged SNORD78 and CEA to predict tumor metastasis in NSCLC

Previous reports presented that both miRNA and lncRNA can act as biomarkers for predicting progression and prognosis. In this study, we were curious about the translation of SNORD in clinical life. Therefore, we further determined the predictive potency of SNORD78 as a biomarker for metastasis. Receiver operating characteristics (ROC) curve was used to evaluate the predictive efficacy of SNORD78. For lymph node metastasis, SNORD78 alone showed low-predictive efficiency since the area under the ROC curve (AUC) was 0.551 (95 % CI 0.372–0.740, p = 0.291, Fig. 8). Next, we explored whether serum tumor biomarkers could improve the predictive efficiency of SNORD78. Among the serum biomarkers analyzed (CEA, AFP, CA125, CA19-9, and NSE), the combination of SNORD78 and CEA significantly improved the predictive efficiency (AUC = 0.642, 95 % CI 0.484–0.791, p = 0.029, Fig. 8).

## Discussion

NSCLC is one the most frequent causes of cancer-related motility. The understanding of NSCLC pathogenesis has improved through the identification of activating mutations in and amplifications of oncogenes, including KRAS [24], EGFR [25], KARS [26], and inactivating mutations in tumor suppressive genes, such as p53 [27, 28]. However, the mechanism of NSCLC development remains largely unknown. A growing volume of literature has indicated the role of small nucleolar RNAs in cancer [7, 9, 11]. snoRNA U50 has been demonstrated to act as a tumor-suppressor in cancer [29, 30]. Mei et al. [7] reported that a diverse number of snoRNAs are differentially expressed in lung cancer with respect to the corresponding matched tissue. Mannoor et al. [15] profiled snoRNA expression in cancer stem cells of lung cancer and found that a number of snoRNAs were significantly upregulated in tumor-initiating cells of lung cancer. SNORD78 was consistently reported to be upregulated in lung cancer [11] and tumor-initiating cells of lung cancer [15], suggesting that SNORD78 might play a role in lung tumorigenesis.

In the present study, we confirmed that the expression of SNORD78 was obviously upregulated in NSCLC tissues compared with adjacent normal tissues and in cancer stem-like cells of NSCLC compared to their non-stem counterparts. Furthermore, SNORD78 was shared in two major histological types of NSCLC. SNORD78 expression was positively correlated with the TNM stage, lymph node metastasis and tumor size. High expression of SNORD78 was also associated with poor prognosis of NSCLC.

We then determine *in vitro* functional significance of SNORD78 in lung cancer cell lines through gain- and loss-of-function analyses. We demonstrated that SNORD78 is required for efficient proliferation and invasion of NSCLC cells. Our data revealed that SNORD78 silencing inhibited cell proliferation via inducing a significant G0/G1 arrest and cell apoptosis. The proliferation-promoting effect of SNORD78 was confirmed with SNORD78 overexpression in A549 cells. SNORD78 silencing suppressed cell invasion via reversing the epithelial-mesenchymal-transition of NSCLC. The concept of cancer stem-like cells or tumor-initiating cells have proposed that the heterogeneous tumor cell population contains a small population of cells with properties such as self-renewal, multiplex differentiation, chemo- and radio-resistance, high tumorigenicity, and they may play pivotal parts in the development, progression, metastasis, recurrence and multidrug resistance of cancer [12, 13]. The identification of molecules that play a role in the self-renewal of cancer stem-like cells may provide a key standpoint for better understanding tumorigenesis and developing prognostic biomarkers and targeted therapy. As SNORD78 is indeed upregulated in cancer stem-like cells of NSCLC, we knocked down SNORD78 in cancer stem-like cells of lung cancer and found that shRNA-SNORD78 transfected cells formed fewer and smaller mammospheres compared with vector-transfected cells, implying that SNORD78 is important for the self-renewal of cancer stem-like cells of NSCLC. Inhibition of SNORD78 resulted in the downregulation of a series of stemness factors, such as Sox2 and Oct4, which has been shown to enhance NSCLC malignancy by inducing cancer stem cell-like properties and epithelial-mesenchymal-transition [25, 26]. The *in vivo* data complement the *in vitro* studies of SNORD78 and confirm the oncogenic activity of SNORD78 in NSCLC.

In conclusion, we demonstrate that the expression of SNORD78 was significantly upregulated in NSCLC tissues. We also showed that SNORD78 promoted the proliferation and invasion of NSCLC cells and is vital for the self-renewal of cancer stem-like cells, suggesting that SNORD78 may play a functional role in NSCLC development. Our study may add our understanding to the molecular mechanisms through which SNORD78 contributes to the tumor progression, which may facilitate the development of snoRNA-directed diagnostics and therapeutics against cancers.
